# *In Vivo* Chemoprotective Activity of Bovine Dialyzable Leukocyte Extract in Mouse Bone Marrow Cells against Damage Induced by 5-Fluorouracil

**DOI:** 10.1155/2016/6942321

**Published:** 2016-04-17

**Authors:** Erika Evangelina Coronado-Cerda, Moisés Armides Franco-Molina, Edgar Mendoza-Gamboa, Heriberto Prado-García, Lydia Guadalupe Rivera-Morales, Pablo Zapata-Benavides, María del Carmen Rodríguez-Salazar, Diana Caballero-Hernandez, Reyes Silvestre Tamez-Guerra, Cristina Rodríguez-Padilla

**Affiliations:** ^1^Laboratorio de Inmunologia y Virologia, Facultad de Ciencias Biológicas, Universidad Autónoma de Nuevo León (UANL), P.O. Box 46 “F”, 66455 San Nicolás de los Garza, NL, Mexico; ^2^Departamento de Enfermedades Crónico-Degenerativas, Instituto Nacional de Enfermedades Respiratorias, Tlalpan 4502, Colonia Sección XVI, 14080 Ciudad de México, DF, Mexico

## Abstract

Chemotherapy treatments induce a number of side effects, such as leukopenia neutropenia, peripheral erythropenia, and thrombocytopenia, affecting the quality of life for cancer patients. 5-Fluorouracil (5-FU) is wieldy used as myeloablative model in mice. The bovine dialyzable leukocyte extract (bDLE) or IMMUNEPOTENT CRP® (ICRP) is an immunomodulatory compound that has antioxidants and anti-inflammatory effects. In order to investigate the chemoprotection effect of ICRP on bone marrow cells in 5-FU treated mice, total bone marrow (BM) cell count, bone marrow colony forming units-granulocyte/macrophage (CFU-GM), cell cycle, immunophenotypification, ROS/superoxide and Nrf2 by flow cytometry, and histological and hematological analyses were performed. Our results demonstrated that ICRP increased BM cell count and CFU-GM number, arrested BM cells in G0/G1 phase, increased the percentage of leukocyte, granulocytic, and erythroid populations, reduced ROS/superoxide formation and Nrf2 activation, and also improved hematological levels and weight gain in 5-FU treated mice. These results suggest that ICRP has a chemoprotective effect against 5-FU in BM cells that can be used in cancer patients.

## 1. Introduction

Most of chemotherapeutic agents can cause myelosuppression in a dose-dependent manner [1]. Other side effects of chemotherapy are alopecia, stomatitis, immunosuppression, anorexia, nausea, and vomiting which result in a decreased functional capacity and quality of life for cancer patients [2]. 5-Fluorouracil (5-FU) is a chemotherapeutic agent used to treat gastrointestinal, breast, pancreatic, and head and neck cancer, among others [3]. The mechanism of cytotoxicity of 5-FU has been ascribed to the misincorporation of fluoronucleotides into RNA and DNA and to the inhibition of the nucleotide synthetic enzyme thymidylate synthase [4]. 5-FU distributes readily into bone marrow, small intestine, kidney, liver, and spleen [5, 6]. In the bone marrow 5-FU, it is incorporated in the DNA and induces oxidative stress, which is partly responsible for myelotoxicity [7, 8]. It is well known that patients treated with 5-FU are cursed with neutropenia, mucositis, leukopenia, and hematological toxicity [9, 10]. Because of these side effects, chemoprotective compounds have been used to reduce these problems [11–19]. Bovine dialyzable leukocyte extract or IMMUNEPOTENT CRP (ICRP) is a dialysate of a heterogeneous mixture of low-molecular-weight substances released from disintegrated leukocytes of the blood or lymphoid tissue obtained from homogenized bovine spleen. ICRP was capable of stimulating the immune system in patients with non-small cell lung cancer and increasing their quality of life [20]. Also,* in vitro* studies demonstrated that ICRP was an effective therapeutic agent in process involving oxidative cellular damage and clinical inflammatory diseases, through I*κ*B/NF-*κ*B pathway [21]. In this study, we examined the protector effect of ICRP on myelosuppression caused by 5-FU in a mouse model.

## 2. Materials and Methods

### 2.1. Animals

Nine-week-old male Balb/c mice were obtained from the bioterium of the Laboratorio de Inmunología y Virología de la Facultad de Ciencias Biológicas. The mice were maintained on pelleted food and water* ad libitum* and housed in controlled environmental conditions (25°C and a 12 h light/dark cycle). The protocol for the animal study was approved by Ethic Review Committee for Animal Experimentation of the Facultad de Ciencias Biológicas, UANL.

### 2.2. Reagents

5-Fluorouracil (5-FU) (Flurox®) was purchased from Lemery (Mexico). N-Acetylcysteine (NAC) was obtained from Sandoz Pharmaceuticals (Mexico). IMMUNEPOTENT CRP (ICRP) was produced by the Laboratorio de Inmunología y Virología, Facultad de Ciencias Biológicas, UANL (San Nicolás de los Garza, Nuevo León, Mexico). ICRP is a low-molecular-weight product (10–12 kDa) from bovine spleen. The extract is dialyzed, lyophilized, and determined as pyrogen-free by* Limulus of amoebocyte* lysate assay (Endotoxin detection kit, ICN Biomedical, Aurora, OH, USA). The ICRP obtained from 1 × 10^8^ leukocytes is defined as one unit (1 U).

### 2.3. Experimental Design

Mice were randomly divided into 5 groups as follows:

 
*Control*: injected* i.p.* on day 0 and* i.m.* for 6 consecutive days with the vehicle (deionized water).
 
*5-FU*: injected* i.p.* with 5-FU in a single dose of 75 mg/kg.
 
*NAC + 5-FU*: injected* i.p.* with NAC in a single dose of 250 mg/kg and one hour later with 5-FU* i.p.* in a single dose of 75 mg/kg as a positive protection control [7].
 ICRP: injected* i.m. *with ICRP (5 U) for 6 consecutive days.
 
*ICRP + 5-FU*: injected* i.m.* with ICRP (5 U), one hour later with 5-FU* i.p*. in a single dose of 75 mg/kg, and for the 6 consecutive days with ICRP (5 U) per day.

The animals were sacrificed on day 1 and day 7 to perform the experiments.

### 2.4. Preparation of Bone Marrow (BM) Cell Suspension

After the mice were sacrificed, both femurs and tibias were dissected. Bone marrow cells were flushed with 5 mL of Iscove's Modified Dulbecco's Medium (IMDM) and supplemented with 2% fetal bovine serum (FBS), antimitotic, and antibiotics. Cells suspensions were centrifuged at 1600 rpm for 10 min and washed twice in IMDM. Final suspension was used for total bone marrow cell count, bone marrow colony forming units-granulocyte/macrophage (CFU-GM) assay, cell cycle, and flow cytometric analysis.

### 2.5. Total BM Cell Count

After the cells were pooled from both femurs and tibias, a count was done by trypan blue exclusion technique, which helps us exclude dead cells from viable cells. To calculate the number of cells obtained from each mouse, 50 *μ*L of the cell suspension was taken and this was transferred to 400 *μ*L of medium plus 50 *μ*L of trypan blue; 10 *μ*L of this suspension was taken and placed in the Neubauer chamber (Bright Line, Reichert, USA). Observation was performed under a microscope at 10x and viable cells present were counted.

### 2.6. CFU-GM Assay

A total of 1 × 10^6^ BM cells were resuspended in 1 mL IMDM supplemented with 2% of FBS, and then 300 *μ*L of this suspension was added to 3 mL of mouse methylcellulose complete media (R&D Systems). Subsequently, the mixture was collected with a 3 mL syringe and 1.1 mL of the final mixture was placed in a 35 mm diameter culture dish; this was done in duplicate. The incubation of dishes was performed according to the manufacturer's instructions. The formation of colonies was observed by microscopy, and the total number of colonies in each dish was counted.

### 2.7. Cell Cycle Analysis

The staining procedures were performed using a BD Cycle Test Plus DNA Reagent Kit according to the instructions of the manufacturer. Cell cycle phase distributions were analyzed in Accuri C6 flow cytometer (BD Biosciences, San Jose, CA). In addition, the percentage of cells in each phase of cell cycle was analyzed by FlowJo software (Treestar, Inc., San Carlos, CA).

### 2.8. Flow Cytometric Analysis

For immunophenotyping, BM cells were stained using fluorescent label-conjugated anti-CD71, anti-ter119, anti-CD45, anti-CD11b, and anti-Gr-1 antibodies (BD Biosciences, San Jose, CA). For intracellular staining, NRF2 (D1Z9C) XP® Rabbit mAb (PE Conjugate) was used, following the technique provided by the manufacturer. For measure of oxidative stress, Total ROS/Superoxide detection kit was used (Enzo Life Sciences). The stained cells were analyzed by Accuri C6 flow cytometer and CFlow plus software (BD Biosciences, San Jose, CA).

### 2.9. Histopathological Analysis

The left femoral bone of each mouse was prepared for general histopathological evaluation, including fixation, decalcification, and sectioning (4 *μ*m thickness), as well as hematoxylin and eosin (HE) staining. Histopathological analysis was done by a veterinarian pathologist.

### 2.10. Hematological Analysis

Blood collection was done by cardiac puncture in EDTA containing vials for immediate analysis of hematological parameters. Hematological analysis was determined by standard clinical procedures using an automatic hematological analyzer (COULTER® Ac·T diff*™* Analyzer, Beckman Coulter).

### 2.11. Weight Gain

Measurement of weight in grams of the mice was performed at the beginning of treatment and seven days later. Weight gain was calculated by subtracting the final weight minus initial weight.

### 2.12. Statistical Analysis

Data was presented as mean ± SD and statistically analyzed using one-way ANOVA test followed by Tukey multiple comparison posttest at *P* < 0.05 (*n* = 3) using SPSS v17 software.

## 3. Results

### 3.1. ICRP Restores the Number and Function of BM Cells in 5-FU Treated Mice

**Figure 1:**
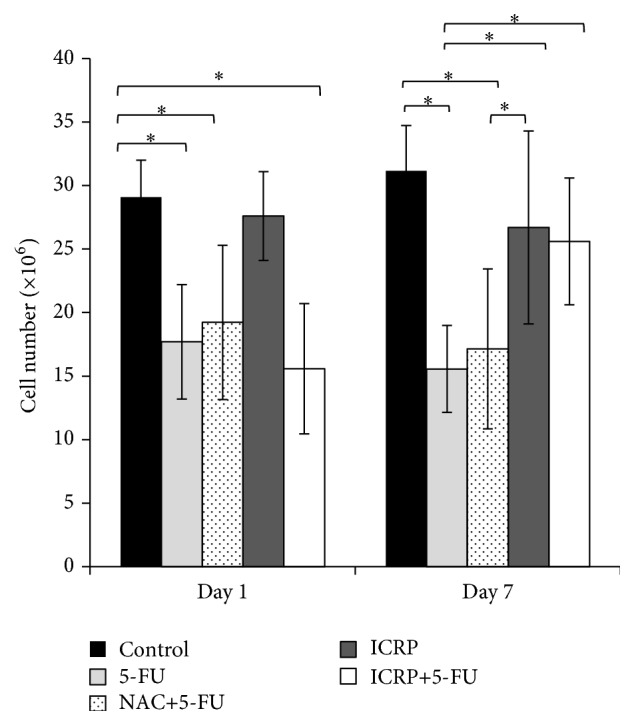
Total bone marrow cell count: BM cells from both femurs and tibias were obtained on day 1 and day 7 from treated mice. Subsequently, BM cells were counted by trypan blue dye exclusion ^*∗*^(*P* < 0.05) (*n* = 3).

The evaluation of the total number of BM cells and the number of CFU-GM was performed 1 and 7 days after the initiating treatment. The number of total BM cells was significantly (*P* < 0.05) decreased in all the groups treated with 5-FU at day 1. Seven days later, the ICRP + 5-FU group showed a recovery compared to 5-FU (*P* < 0.05) and NAC + 5-FU groups. On the other hand, the use of ICRP treatment by itself did not change, compared to the control (Figure 1).

**Figure 2:**
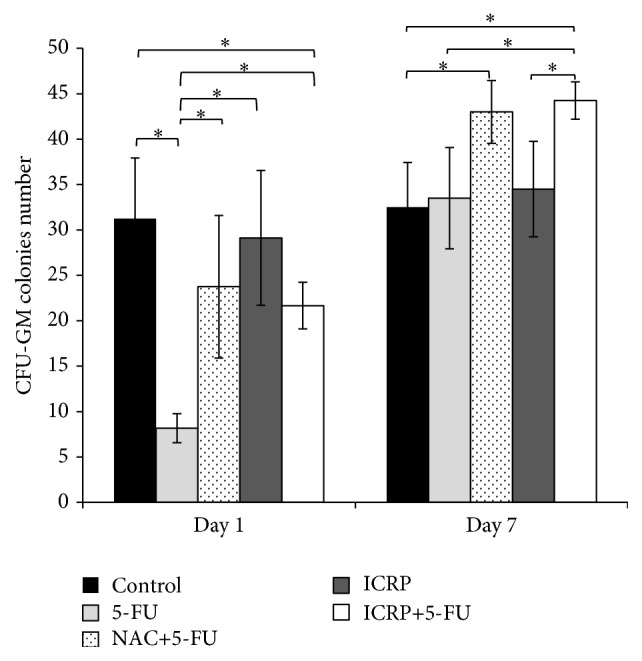
Colony forming units-granulocyte/macrophage (CFU-GM) assay: BM cells from both femurs and tibias were obtained on day 1 and day 7 from treated mice. Subsequently, BM cells were grown mouse methylcellulose complete media in a 5% CO_2_ incubator for 14 days and colonies were counted ^*∗*^(*P* < 0.05) (*n* = 3).

When the evaluation of the number of CFU-GM was done, we observed that ICRP and NAC treatments reversed the side effects of 5-FU related to a decrease in the number of CFU-GM colonies (*P* < 0.05) on day 1; seven days later, NAC + 5-FU and ICRP + 5-FU groups increased the number of CFU-GM compared to the control (Figure 2).

### 3.2. ICRP Does Not Affect Cell Cycle Phases on BM Cells in 5-FU Treated Mice

Treatment with 5-FU significantly decreased S phase and NAC and ICRP treatments did not change this effect on BM cells. The 5-FU group increased the percentage of Sub-G1 phase, which indicates that cells are under apoptosis, on day 1. The cell cycle was not affected (*P* < 0.05) by treatments on day 7 (Table 1).

### 3.3. ICRP Restores Leukocyte, Granulocyte, and Erythrocyte Populations in BM Cells of 5-FU Treated Mice

**Figure 3:**
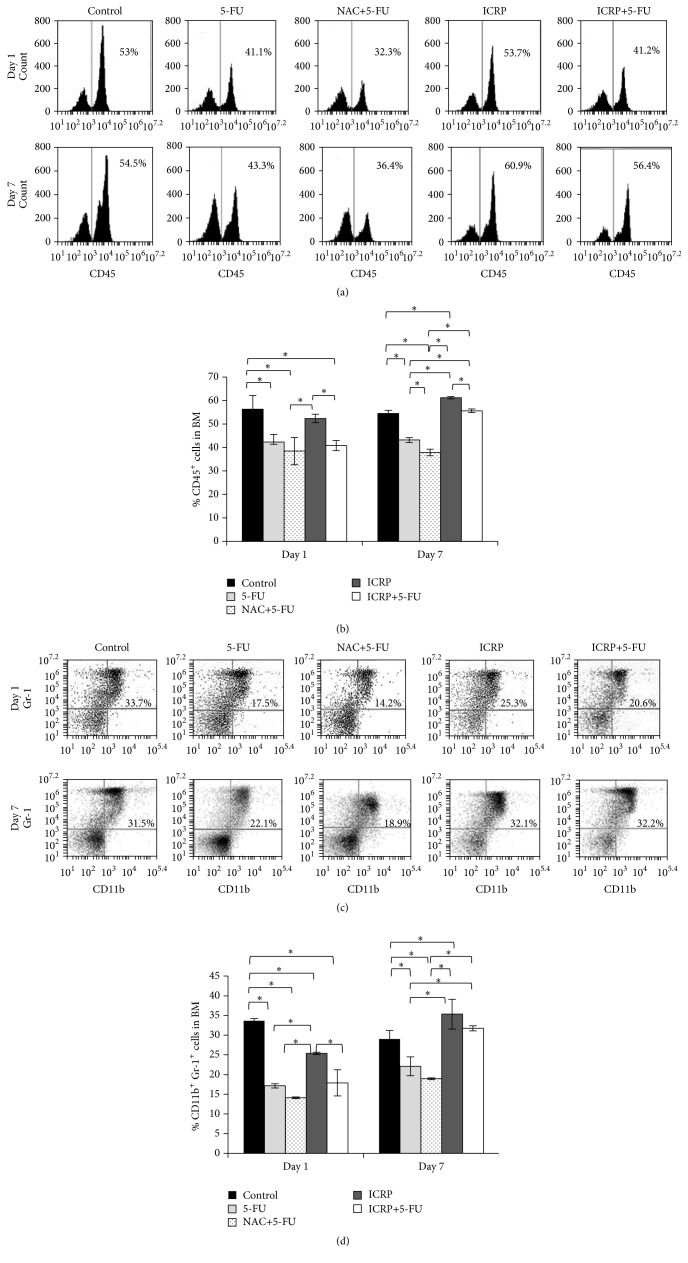
Flow cytometry analysis: BM cells from both femora and tibia were obtained on day 7 from treated mice. Subsequently, BM cells were analyzed for expression of the cell surface markers by flow cytometry. (a) Representative result of flow cytometry analysis for CD45^+^ BM cells. (b) Statistics from CD45^+^ BM cells. (c) Representative result of flow cytometry analysis for CD11b^+^Gr-1^+^ BM cells. (d) Statistics from CD11b^+^Gr-1^+^ BM ^*∗*^(*P* < 0.05) (*n* = 3).

To elucidate the specific population that is protected by the ICRP, the percentages of leukocyte (CD45^+^), granulocytic (CD11b^+^Gr-1^+^), and erythroid (CD71, Ter119) lineages in BM cells were evaluated by flow cytometry. On day one, leukocyte and granulocytic populations were decreased (*P* < 0.05) by 5-FU treatment; NAC and ICRP did not protect BM cells of 5-FU treated mice. The 5-FU group evaluated on day 7 kept low percentages of CD45^+^ and CD11b^+^Gr-1^+^ populations but ICRP + 5-FU group increased these populations (*P* < 0.05) similar to the control group (Figure 3).

**Figure 4:**
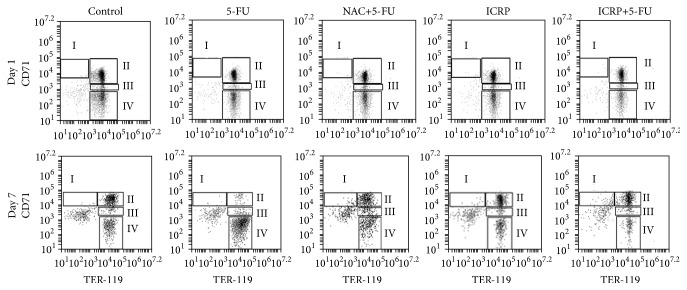
Flow cytometry analysis: representative result of flow cytometry analysis. BM cells from both femurs and tibias were obtained on day 7 from treated mice. Subsequently, BM cells were analyzed for expression of the cell surface markers CD71 and Ter119 by flow cytometry. Populations I to IV represent progressive maturation of erythroid cells. Populations are characterized by I, proerythroblasts; II, basophilic erythroblasts; III, orthochromatic erythroblast; and IV, enucleated red blood cells.

The protective effect of ICRP on erythroid lineage was evident on days 1 and 7, because ICRP + 5-FU group preserves highest percentages in basophilic erythroblast and orthochromatic erythroblast stages of erythroid maturation compared with 5-FU group (*P* < 0.05). Enucleated red blood cells were the predominant population in 5-FU treated mice; these findings are different to the control and ICRP + 5-FU groups (Figure 4 and Table 2).

### 3.4. ICRP Decreased ROS/Superoxide Formation and Nrf2 Activation Induced by 5-FU

**Figure 5:**
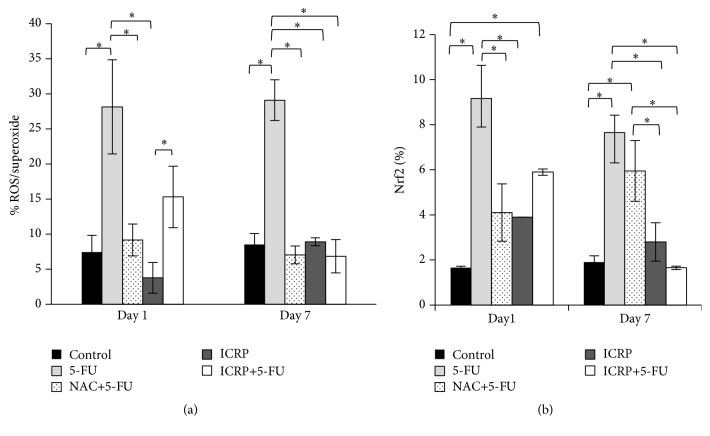
(a) ROS/superoxide analysis: BM cells from both femora and tibia were obtained on day 1 and day 7 from treated mice. Subsequently, BM cells were stained for ROS/superoxide detection by flow cytometry. (b) Nrf2 analysis: BM cells from both femora and tibia were obtained on day 1 and day 7 from treated mice. Subsequently, BM cells were fixed and permeabilized for intracellular staining ^*∗*^(*P* < 0.05) (*n* = 3).

The ROS/superoxide formation and Nrf2 activation were induced on day 1 and day 7 by 5-FU treatment and the ICRP + 5-FU treatment decreases these parameters on day 7; no statistical difference was found on day 1 (Figure 5).

### 3.5. Histopathologic Analysis

**Figure 6:**
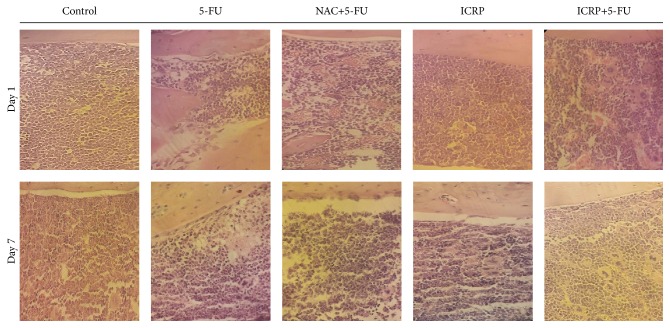
Bone marrow histology: on day 1 and day 7, femora of treated mice were harvested, fixed, sectioned, and stained with hematoxylin and eosin. The histological description was made by a pathologist; photographs were taken under a microscope at 40x.

The effect of ICRP on the histopathology of bone marrow at 1 and 7 days is shown in Figure 6 and described at continuation; 5-FU treatment decreased the cell density of bone marrow, creating a hypocellular environment, with marked decrease of megakaryocytes and granulocytic lineage cells, and no large amount of precursor cells was found. NAC and ICRP treatments protect bone marrow because they present a higher proportion in differentiated precursor cells and cellularity. The ICRP group showed normal myeloid tissue cellularity.

### 3.6. Effects of ICRP on Hematotoxicity and Gain of Body Weight in 5-FU Treated Mice

We examined the effects of ICRP on 5-FU treated mice on red blood cell (RBC), hemoglobin (HB), hematocrit (HCT), white blood cell (WBC), and platelets (PLT) levels 1 and 7 days after initiating treatments. The 5-FU group resulted in anemia and erythrocytopenia and decreased the hematocrit level on day 1 and day 7. The leucopenia was observed only in day 1. The ICRP + 5-FU group did not present any of these toxic effects; their values were similar to the control group (Table 3). The 5-FU treated mice gained less body weight (*P* < 0.05) compared to the control. The ICRP + 5-FU group increased body weight similar to the control group (Table 4).

## 4. Discussion

The chemotherapy with 5-FU is widely used since its discovery to treat a variety of tumors, including colorectal, breast, and liver carcinomas [22]. The hematologic toxicity induced by chemotherapy is related to the dose-limiting side effect, affecting the therapeutic success and quality of life of patients [23]. The 5-FU as a model of bone marrow depletion has been used by many researchers [7, 13, 16, 17, 24–28]; in the present study, we corroborated that a single dose of 5-FU (75 mg/kg) reduces the number of CFU-GM [7], which indicates that bone marrow lineage commitment and proliferative potential are affected [29]. The ICRP treatment demonstrates an efficient chemoprotection to 5-FU treatment, due to an increase in bone marrow progenitor cells function, such as those found with the use of amifostine, which is a clinical radioprotector [30].

This effect on progenitor cells can be correlated with the capacity of ICRP to protect more committed lineages in bone marrow cells, such as leukocyte (CD45^+^), granulocyte (CD11b^+^Gr-1^+^), and erythroid populations (CD71, Ter119) which are affected by 5-FU [27, 31], and also with normal hematological values of WBC and RBC in a systemic level. Other studies have previously used animal models and peripheral blood reconstitution as measure of bone marrow recovery after chemotherapy [32, 33]. These results could be used to reduce infections and anemia experienced by patients receiving chemotherapy [34].

In this study, cell cycle analysis was used to determine whether the observed chemoprotection is related to cell cycle arrest at any phase. It is known that the mechanism of cytotoxicity of 5-FU is on actively proliferating cells (S phase) including healthy and cancer cells [35, 36]. Agents such as tetrapeptide acetyl-N-Ser-Asp-Lys-Pro (AcSDKP) and TGF-*β* can protect marrow progenitor cells due to the induction of G0/G1 arrest, being an alternative to chemoprotection therapy [37, 38]. In our study, ICRP and NAC did not affect cell cycle in bone marrow cells, suggesting another mechanism of action in the protection of 5-FU treated bone marrow cells.

It is know that 5-FU induces oxidative stress in bone marrow cells [7] and this leads to the activation of Nrf2 transcription factor [39, 40]; therefore, several researchers explain the mechanism of chemoprotection of different compounds for their ability to activate the antioxidant response and neutralizing ROS [41]. Our results indicate that ICRP decreased ROS/superoxide production and Nrf2 activation in 5-FU treated bone marrow cells on 7 day; this could be explained because ICRP has an antioxidant capacity by increasing glutathione peroxidase, catalase, and superoxide dismutase enzymes [21]; further studies are needed to clarify whether these enzymes are responsible for decreasing ROS production in bone marrow. These results would suggest that ICRP might act as free radical scavenger, similar to aminothiols and phosphorothioates, two protective agents widely used [42].

All these effects of chemoprotection are reflected in our histopathology analysis; this kind of technique is used to assess the bone marrow architecture, cellularity, estimation of iron stores, and other features [43]. It is important that patients receiving chemotherapy can maintain their weight in order to improve health-related quality of life [44]; we found that ICRP helps to maintain normal weight after 5-FU treatment, which indicates that ICRP could improve the quality of life in cancer patients. It is known that NAC protects the cytotoxic and apoptotic effects against cisplatin in human tumor cell lines, because NAC blocks the death receptor and mitochondrial apoptotic pathways [45]. This is necessary in* in vitro* and* in vivo* studies to determinate if ICRP has antagonist action against tumor cells treated with chemotherapy due to its antioxidant activity showed in this study. Current studies about that are running in our laboratory. Although in previous studies ICRP has been administrated to patients with breast and lung cancer as an adjuvant to avoid secondary effects in combination with chemotherapy, there was no effect on the tumor regression and improving the quality of life [20, 46].

## 5. Conclusion

It is important to investigate new compounds that could be given during chemotherapy treatment and help us to alleviate some side effects, resulting in a significant increase in chemotherapy doses. Our results suggest that the ICRP can be proposed as a chemoprotective agent because it is able to protect the damage caused by 5-FU in bone marrow cells, ROS production, hematological parameters, and weight gain probably by its antioxidant or immunomodulatory capacity.

## Figures and Tables

**Table 1:** Effects of 5-FU, NAC + 5-FU, ICRP, and ICRP + 5-FU treatments on cell cycle phases in mice.

Cell cycle phases%					
	Groups	G0/G1	S	G2/M	Sub-G1
Day 1	Control	71.97 ± 2.80	21.27 ± 1.93	4.32 ± 0.77	2.00 ± 0.27
	5-FU	85.13 ± 3.25^a^	7.92 ± 1.03^a^	3.21 ± 1.06	4.10 ± 1.34^a^
	NAC + 5-FU	86.10 ± 1.93^a^	7.40 ± 0.15^a^	3.18 ± 0.52	3.67 ± 0.57
	ICRP	71.33 ± 2.26^b,c^	21.67 ± 1.46^b,c^	4.17 ± 1.31	3.20 ± 0.30
	ICRP + 5-FU	89.53 ± 0.31^a,d^	6.15 ± 0.75^a,d^	2.86 ± 0.89	3.19 ± 0.99

Day 7	Control	79.27 ± 1.95	15.87 ± 1.80	4.94 ± 1.21	2.51 ± 1.09
	5-FU	66.57 ± 11.05	22.47 ± 6.76	5.82 ± 1.57	7.37 ± 3.48
	NAC + 5-FU	70.30 ± 0.95	25.03 ± 0.70	6.56 ± 2.53	2.12 ± 0.73
	ICRP	73.80 ± 7.71	18.27 ± 3.95	8.17 ± 4.09	4.69 ± 1.65
	ICRP + 5-FU	67.40 ± 2.72	21.70 ± 4.52	8.34 ± 5.52	5.12 ± 3.63

Data are expressed as mean ± SD (*P* < 0.05) (*n* = 3).

a: significantly different from the control group.

b: significantly different from 5-FU group.

c: significantly different from NAC + 5-FU group.

d: significantly different from ICRP group.

**Table 2:** Effects of 5-FU, NAC + 5-FU, ICRP, and ICRP + 5-FU on erythrocyte population in treated mice.

	Erythrocyte population%				
	Groups	I Proerythroblasts	II Basophilic erythroblasts	III Orthochromatic erythroblast	IV Enucleated red blood cells
Day 1	Control	0.53 ± 0.06	49.00 ± 2.65	6.60 ± 0.46	25.17 ± 1.07
	5-FU	0.07 ± 0.12^a^	40.07 ± 4.56^a^	3.50 ± 0.20^a^	31.03 ± 2.57^a^
	NAC + 5-FU	0.23 ± 0.06	39.67 ± 3.28^a^	5.50 ± 0.30^a,b^	26.93 ± 2.93
	ICRP	0.30 ± 0.00^b^	51.80 ± 0.36^b,c^	6.50 ± 0.30^b,c^	20.53 ± 0.45^b,c^
	ICRP + 5-FU	0.20 ± 0.10	57.03 ± 2.59^b,c^	4.20 ± 0.26^a,c,d^	20.20 ± 1.55^a,b,c^

Day 7	Control	0.33 ± 0.05	48.3 ± 1.95	3.10 ± 0.87	20.57 ± 2.48
	5-FU	0.27 ± 0.11	1.70 ± 0.26^a^	3.50 ± 0.88	71.87 ± 4.48^a^
	NAC + 5-FU	4.40 ± 2.40^a,b^	27.3 ± 5.66^b^	6.67 ± 1.52^a,b^	21.63 ± 3.3^b^
	ICRP	0.63 ± 0.05^c^	51.9 ± 3.93^b,c^	4.90 ± 0.40	20.77 ± 4.31^b^
	ICRP + 5-FU	4.13 ± 0.77^a,b,d^	52.1 ± .59^b,c^	6.83 ± 0.70^a,b^	16.30 ± 1.57^b^

Data are expressed as mean ± SD (*P* < 0.05) (*n* = 3).

a: significantly different from the control group.

b: significantly different from 5-FU group.

c: significantly different from NAC + 5-FU group.

d: significantly different from ICRP group.

Populations I to IV represent progressive maturation of erythroid cells. Populations are characterized by I, proerythroblasts; II, basophilic erythroblasts; III, orthochromatic erythroblast; and IV, enucleated red blood cells.

**Table 3:** Effects of 5-FU, NAC + 5-FU, ICRP, and ICRP + 5-FU on peripheral blood analysis in treated mice.

Peripheral blood analysis						
	Group	RBC count × 10^6^	HB level g/dL	HCT level (%)	WBC mm^3^	PLT mm^3^
Day 1	Control	8.00 ± 0.35	12.50 ± 0.85	36 ± 4	4.67 ± 0.49	534.33 ± 170.28
	5-FU	4.43 ± 0.31^a^	6.87 ± 0.61^a^	20 ± 2^a^	1.50 ± 0.44^a^	318.33 ± 123.31
	NAC + 5-FU	6.97 ± 0.81^b^	11.30 ± 1.39^b^	32 ± 5^b^	2.23 ± 0.85	393.00 ± 54.29
	ICRP	8.30 ± 0.44^b^	13.10 ± 0.72^b^	39 ± 2^b^	6.37 ± 1.77^b,c^	448.67 ± 108.84
	ICRP + 5-FU	6.90 ± 1.14^b^	11.03 ± 1.61^b^	32 ± 5^b^	4.13 ± 0.91	472.33 ± 159.53

Day 7	Control	7.60 ± 0.92	11.63 ± 1.65	34 ± 6	5.03 ± 0.95	525.00 ± 184.01
	5-FU	4.83 ± 1.27^a^	7.73 ± 2.48^a^	22 ± 7	3.13 ± 1.90	260.67 ± 122.92
	NAC + 5-FU	6.93 ± 0.59^b^	10.50 ± 0.95	32 ± 3	5.60 ± 3.74	380.00 ± 34.18
	ICRP	8.63 ± 0.15^b^	13.43 ± 0.50^b^	42 ± 5^b^	6.27 ± 1.87	470.67 ± 82.78
	ICRP + 5-FU	7.73 ± 0.35^b^	12.17 ± 0.45^b^	36 ± 2^b^	4.57 ± 1.10	600.00 ± 89.21^b^

Data are expressed as mean ± SD (*P* < 0.05) (*n* = 3).

a: significantly different from the control group.

b: significantly different from 5-FU group.

c: significantly different from NAC + 5-FU group.

Red blood cell (RBC), hemoglobin (HB), hematocrit (HCT), white blood cell (WBC), and platelets (PLT).

**Table 4:** Effects of 5-FU, NAC + 5-FU, ICRP, and ICRP + 5-FU on body weight gain in treated mice.

Body weight			
Group	Initial body weight (g)	Final body weight (g)	Body weight gain (g)
Control	26.50 ± 3.00	29.75 ± 2.63	3.25 ± 0.50
5-FU	24.00 ± 4.62	25.25 ± 4.35	1.25 ± 0.50^a^
NAC + 5-FU	23.50 ± 5.20	25.25 ± 5.06	1.75 ± 0.50^a^
ICRP	25.50 ± 4.51	29.00 ± 4.97	3.50 ± 0.58^b,c^
ICRP + 5-FU	25.75 ± 3.59	28.75 ± 4.03	3.00 ± 0.82^b^

Data are expressed as mean ± SD (*P* < 0.05) (*n* = 3).

a: significantly different from the control group.

b: significantly different from 5-FU group.

c: significantly different from NAC + 5-FU group.
